# European Academy of Nursing Science 2016 Summer Conference: Halle, Germany. 13-14 July 2016

**DOI:** 10.1186/s12912-016-0186-y

**Published:** 2016-12-06

**Authors:** Walter Sermeus, Nicky Cullum, Katrin Balzer, Rhian Schröder, Anne Junghans, Ute Stahl, Jens-Martin Träder, Sascha Köpke, Martin N. Dichter, Rebecca Palm, Margareta Halek, Sabine Bartholomeyczik, Gabriele Meyer, Daniela Holle, Rabea Graf, Ute Rosier, Sven Reuther, Martina Roes, Margareta Halek, Bruna R. Gouveia, Helena G. Jardim, Maria M. Martins, Duarte L. Freitas, José A. Maia, Debra J. Rose, Élvio R. Gouveia, Luk Bruyneel, Emmanuel Lesaffre, Walter Sermeus, J. E. Ball, L. Bruyneel, L. Aiken, C. Tishelman, W. Sermeus, P. Griffiths, Evridiki Papastavrou, Panayiota Andreou, Loredana Sasso, Annamaria Bagnasco, Milko P. Zanini, Gianluca Catania, Giuseppe Aleo, Federico Spandonaro, Giancarlo Icardi, Roger Watson, Walter Sermeus, Steffen Fleischer, Marion Burckhardt, Gabriele Meyer, Almuth Berg, Ann Van Hecke, Simon Malfait, Johan Van Daele, Kristof Eeckloo, Mieke Deschodt, Bastiaan Van Grootven, Koen Milisen, Johan Flamaing, Anne C. Rahn, Sascha Köpke, Imke Backhus, Jürgen Kasper, Anna Krützelmann, Ingo Kleiter, Ingrid Mühlhauser, Christoph Heesen

**Affiliations:** 1 Leuven Institute for Healthcare Policy, Department of Public Health & Primary Care, KU Leuven, Leuven, Belgium; 2 School of Nursing, Midwifery & Social Work, University of Manchester, Manchester, UK and Research and Innovation, Central Manchester University Hospitals NHS Foundation Trust, Grafton Street, Manchester, M13 9WU UK; 3 Nursing Research Unit, Institute for Social Medicine and Epidemiology, University of Lübeck, Lübeck, Schleswig-Holstein 23538 Germany; 4 Department of Nursing and Management, University of Applied Sciences Hamburg, Hamburg, 20099 Germany; 5 AMEOS Krankenhausgesellschaft, Neustadt, Schleswig-Holstein 23730 Germany; 6 Institute for Family Medicine, University of Lübeck, Lübeck, Schleswig-Holstein 23538 Germany; 7 German Center for Neurodegenerative Diseases (DZNE), Stockumer Straße 12, 58453 Witten, Germany; 8 School of Nursing Science, Witten/Herdecke University, Stockumer Straße 12, 58453 Witten, Germany; 9 Institute for Health and Nursing Science, Medical Faculty, Martin Luther University, Halle-Wittenberg, Germany; 10 German Center of Neurodegenerative Diseases (DZNE), Witten, 58453 Witten, Germany; 11 School of Nursing Science, Witten/Herdecke University, Witten, 58453 Witten, Germany; 12 Saint Joseph of Cluny Higher School of Nursing, Funchal, 9050-535 Portugal; 13 Institute of Biomedical Sciences Abel Salazar, University of Porto, Porto, 4050-313 Portugal; 14 Higher School of Health, University of Madeira, Funchal, 9000-082 Portugal; 15 Higher School of Nursing of Porto, Porto, 4200-072 Portugal; 16 Department of Physical Education and Sports, University of Madeira, Funchal, 9000-082 Portugal; 17 Faculty of Sport, University of Porto, Porto, 4200-450 Portugal; 18 Division of Kinesiology and Health Science, California State University Fullerton, Fullerton, California 92831-3599 USA; 19 Leuven Institute for Healthcare Policy, KU Leuven – University of Leuven, 3000 Leuven, Belgium; 20 Leuven Biostatistics and Statistical Bioinformatics Centre (L-BioStat), KU Leuven – University of Leuven, 3000 Leuven, Belgium; 21 Faculty of Health Sciences, University of Southampton UK & National Institute for Health Research Collaboration for Leadership in Applied Health Research and Care (CLAHRC) Wessex, Southampton, UK; 22 Leuven Institute for Healthcare Policy, KU Leuven – University of Leuven, 3000 Leuven, Belgium; 23 Medical Management Centre (MMC), Department of Learning, Informatics, Management and Ethics (LIME), Karolinska Institutet (KI), Stockholm, Sweden; 24 Center for Health Outcomes and Policy Research, University of Pennsylvania School of Nursing, Philadelphia, PA 19104 USA; 25 Department of Nursing, Cyprus University of Technology, Limassol, 3011 Cyprus; 26 Department of Health Sciences, University of Genoa, Genoa, 16132 Italy; 27 Faculty of Economics, University of Rome Tor Vergata, Rome, 00133 Italy; 28 Faculty of Health and Social Care, University of Hull, Hull, HU6 7RX UK; 29 Department of Public Health and Primary Care, KU Leuven, Leuven, 3000 Belgium; 30 Institute of Nursing and Health Sciences, Medical Faculty, Martin Luther University Halle-Wittenberg, Halle (Saale), 06112 Germany; 31 Faculty of Medicine and Health Sciences, University Centre for Nursing and Midwifery, Ghent University, 9000 Ghent, Belgium; 32 Ghent University Hospital, 9000 Ghent, Belgium; 33 Department of Public Health and Primary Care, University of Leuven KU Leuven, Leuven, Belgium; 34 Department of Clinical and Experimental Medicine, University of Leuven KU Leuven, Leuven, Belgium; 35 Department of Public Health, University of Basel, Basel, Switzerland; 36 Division of Geriatrics, University Hospitals Leuven, Leuven, Belgium; 37 Unit of Health Sciences and Education, University of Hamburg, Hamburg, 20146 Germany; 38 Institute for Neuroimmunology and Multiple Sclerosis, Department of Neurology, University Medical Centre Hamburg-Eppendorf, Hamburg, 20246 Germany; 39 Nursing Research Unit, Institute of Social Medicine and Epidemiology, University of Lübeck, Lübeck, Schlesweig-Holstein 23538 Germany; 40 Department of Health and Care Sciences, University of Tromsø, Tromsø, 9019 Norway; 41 Ruhr-University Bochum, St. Josef Hospital, Bochum, 44791 Germany

## KEYNOTE PRESENTATIONS

### K1 Understanding the role of nurses in patient safety: from evidence to policy with RN4CAST

#### Walter Sermeus

##### Leuven Institute for Healthcare Policy, Department of Public Health & Primary Care, KU Leuven, Leuven, Belgium

**Background**

Nurses have a twofold role in healthcare. On one hand, they provide care to patients who are not able to take care of themselves. On the other hand nurses play a vital role as guardians of patient safety. It is mainly on the second role of nurses that the EU funded RN4CAST project is focusing.

**Materials and methods**

Data from more than 33,000 nurses and 11,000 patients in 500 hospitals from 12 European countries were collected from 2009 until 2011. It is one of the largest databases in the world on nurses staffing and its impact on patient safety.

**Results**

One of the main results [1] is that an increase in a nurses’ workload by one patient increases the likelihood of an inpatient dying within 30 days of admission by 7%. And every 10% increase in bachelor’s degree nurses is associated with a decrease in this likelihood by 7%. Relating this finding to the human error theory of James Reason, there seem to be a knowledge problem in early detecting of risks and adverse events. Secondly there are some slips and lapses in execution because of a too high workload. As an example, one out of four nurses say that they didn’t have the time to perform proper patient surveillance [2]. Again, this is explained by nurse staffing, education, working environment and non-nursing tasks to be performed.

**Discussion and conclusions**

In the follow-up of the report of the Institute of Medicine on “to err is human” [3], the crucial role of nurses in patient safety is highly underestimated [4]. This is mainly because the evidence is recent and mainly based on observational data. But the evidence is growing and consistent and strong evaluated against the Bradford-Hill criteria of causation (1965), showing that mechanisms are well understood, can be replicated across populations and settings and are time and dose-response related. Based on the available evidence, we see that some countries are adapting already their legislation to provide safe nurse staffing ratios to create a safe environment for their patients.

**Acknowledgment**

The study was financed by the European Union’s Seventh Framework Programme (FP7/2007–2013, grant agreement no. 223468.

**References**

1. Aiken LH, Sloane DM, Bruyneel L, Van den Heede K, Griffiths P, Busse R, Diomidous M, Kinnunen J, Kózka M, Lesaffre E, McHugh MD, Moreno-Casbas MT, Rafferty AM, Schwendimann R, Scott PA, Tishelman C, van Achterberg T, Sermeus W, Nurse staffing and education and hospital mortality in nine European countries: a retrospective observational study, The Lancet 26 February 2014.

2. Ausserhofer D, Zander B, Busse R, Schubert M, De Geest S, Rafferty AM, Ball J, Scott A, Kinnunen J, Heinen M, Strømseng Sjetne I, Moreno-Casbas T, Kózka M, Lindqvist R, Diomidous M, Bruyneel L, Sermeus W, Aiken LH, Schwendimann R; on behalf of the RN4CAST consortium. Prevalence, patterns and predictors of nursing care left undone in European hospitals: results from the multicountry cross-sectional RN4CAST study. BMJ Qual Saf. 2013 Nov 10.

3. Kohn LT, Corrigan JM, Donaldson MS, editors. To Err is Human: Building a Safer Health System. Washington (DC): National Academies Press (US); 2000.

4. Wachter RM. Patient safety at ten: unmistakable progress, troubling gaps. Health Aff (Millwood). 2010 Jan-Feb;29(1):165–73.

### K2 Research: what can nursing learn from wounds?

#### Nicky Cullum

##### School of Nursing, Midwifery & Social Work, University of Manchester, Manchester, UK and Research and Innovation, Central Manchester University Hospitals NHS Foundation Trust, Grafton Street, Manchester M13 9WU, UK

Complex wounds affect approximately 1.5 people per 1000, impair quality of life and are costly to health services. In the UK community nurses lead the care of people with complex wounds, from assessment to treatment selection. Some of the earliest randomised controlled trials (RCTs) of wound treatments were of oral zinc. Published in the 1970s these trials used different doses of zinc, measured different outcomes, had different eligibility criteria and very small sample sizes. Each new trial failed to build on the evidence that already existed or learned from mistakes. When a systematic review of the evidence for oral zinc was finally conducted, it concluded that we have no idea whether it makes a difference and the evidence is very low quality [1].

Systematic reviews should be the starting point for all new research. Cochrane reviews provide a lens for scrutiny of the quality of wounds trials and the evolution of the evidence. All Cochrane reviews appraise the included studies in a standardised way. The 199 RCTs included in the five largest Cochrane Wounds reviews have a median sample size of only 115, and most are at high risk of bias (due to unmasked outcome assessment, unconcealed allocation or poor randomisation technique) or the risk of bias is unclear due to poor reporting. Happily there is some evidence that study quality is improving over time. There is plentiful evidence that wounds research teams often lack methodological expertise (e.g., in statistics and trials). Nursing research can and should avoid these pitfalls and build evidence that can be *used* because, in the case of RCTs, there is an adequate sample size for the question and the RCT is designed to minimise bias.

One of the main reasons for undertaking systematic reviews is to identify gaps in the evidence so that new research can fill them. A positive impact of systematic reviews in wound care is that they have stimulated research funders to commission high quality research to fill the gaps, particularly in the UK where the National Institute for Health Research is one of the world’s largest independent funders of wounds research [2-4].

Finally wounds research has failed to develop a basic theoretical understanding. For example one reason why we do not have many effective treatments for complex wounds is because we do not fully understand their aetiology and prognostic factors so do not target treatments effectively. Again nursing research can avoid this mistake by ensuring indepth theoretical knowledge of the health problem concerned and such an understanding is fundamental to developing complex interventions.

**References**

1. Wilkinson EAJ. Oral zinc for arterial and venous leg ulcers. Cochrane Database of Systematic Reviews 2014, Issue 9. Art. No.: CD001273. doi:10.1002/14651858.CD001273.pub3.

2. Michaels JA, Campbell WB, King BM, Macintyre J, Palfreyman SJ, Shackley P, et al. A prospective randomised controlled trial and economic modelling of antimicrobial silver dressings versus non-adherent control dressings for venous leg ulcers: The VULCAN trial. Health Technol Assess 2009;13(56).

3. Ashby RL, Gabe R, Ali S, Saramago P, Chuang LH, Adderley U, et al. VenUS IV (Venous leg Ulcer Study IV) - compression hosiery compared with compression bandaging in the treatment of venous leg ulcers: a randomised controlled trial, mixed-treatment comparison and decision-analytic model. Health Technol Assess 2014;18(57).

4. Nixon J, Cranny G, Iglesias C, Nelson EA, Hawkins K, Phillips A et al. Randomised, controlled trial of alternating pressure mattresses compared with alternating pressure overlays for the prevention of pressure ulcers: PRESSURE (pressure relieving support surfaces) trial BMJ 2006;332:1413.

## SPEAKER PRESENTATIONS

### S1 Dementia care training in undergraduate nursing education: Lessons learned from a pilot study on a novel inter-professional training course

#### Katrin Balzer^1^, Rhian Schröder^1^, Anne Junghans^2^, Ute Stahl^3^, Jens-Martin Träder^4^, Sascha Köpke^1^

##### ^1^Nursing Research Unit, Institute for Social Medicine and Epidemiology, University of Lübeck, Lübeck, Schleswig-Holstein, 23538, Germany; ^2^Department of Nursing and Management, University of Applied Sciences Hamburg, Hamburg, 20099, Germany; ^3^AMEOS Krankenhausgesellschaft, Neustadt, Schleswig-Holstein, 23730, Germany; ^4^Institute for Family Medicine, University of Lübeck, Lübeck, Schleswig-Holstein, 23538, Germany

###### **Correspondence:** Katrin Balzer (katrin.balzer@uksh.de)

**Background**

Insufficient knowledge among nurses and medical doctors regarding evidence-based care recommendations and nurse-doctor-communication failures are assumed to be important determinants of malpractices in dementia care. To address these barriers early in the professionals’ careers, an inter-professional dementia care training course was developed for undergraduate nursing and medical students.

**Aim**

To explore the feasibility of the novel course and potential effects on participants’ knowledge and attitudes.

**Methods**

A before-after study was conducted alongside preliminary course implementation in three terms. All participants were asked to complete a questionnaire at the beginning and the end of the course. The questionnaires covered following outcome areas: various dimensions of the course’s process quality, including relevance of the inter-professional learning, and knowledge and attitudes related to multiple aspects of dementia care. Data were descriptively analysed.

**Results**

In the first term, the course had to be terminated early due to dominating barriers making full implementation not feasible. Across the following terms, the then revised course was attended by 15 nursing and 17 medical students, who all participated in the pilot study. The overall course quality was rated with “2” on a six-step rating scale (1 = very good, 6 = insufficient). The inter-professional course character was highly valued both by the nursing and the medical students. However, differences between these two sub-samples were observed with regard to the assessment of other course aspects (e.g. problem-based learning) and the changes in students’ knowledge and attitudes.

**Conclusions**

Due to the mixed findings, further revision of the curriculum is required, taking the experiences from this pilot study into account.

**Relevance for nursing science in Europe**

This study exemplarily shows how nursing education can be developed further by means of complex intervention research inventory.

### S2 The perspective of people with dementia: Influencing factors of dementia-specific Quality of Life

#### Martin N. Dichter^1,2^, Rebecca Palm^1,2^, Margareta Halek^1,2^, Sabine Bartholomeyczik^2^, Gabriele Meyer^3,2^

##### ^1^German Center for Neurodegenerative Diseases (DZNE), Stockumer Straße 12, 58453 Witten, Germany; ^2^School of Nursing Science, Witten/Herdecke University, Stockumer Straße 12, 58453 Witten, Germany; ^3^Institute for Health and Nursing Science, Medical Faculty, Martin Luther University, Halle-Wittenberg, Germany

###### **Correspondence:** Martin N. Dichter (Martin.Dichter@dzne.de)

**Background**

Despite missing theoretical clarity, quality of life (Qol) has become a major concept as outcome in intervention studies in dementia research. There is no generally accepted definition of Qol for people with dementia. Lawton’s model (1991), which includes subjective and objective components, is widely used as framework for the development of Qol measurements and interventions. The aim of the study is the identification of factors that affect dementia-specific quality of life (Qol).

**Material and methods**

The meta-synthesis (PROSPERO 2013:CRD 42013005014) followed four methodological steps: (a) database search without time limit including forward and backward citation tracking, (b) data extraction, (c) quality appraisal using formal criteria from the Critical Appraisal Skills Programme, (d) synthesis of findings based on principles and procedures of grounded theory. In particular, the constant comparative method leads the coding, identification of categories and synthesis. Two independent reviewers carried out all four methodological steps.

**Results**

The literature search and removal of duplicates revealed 3475 abstracts; 63 full texts were screened for eligibility. Eleven studies from seven countries, published between 1996 and 2011, were included. The papers comprised reports on the perspectives of 410 people with dementia in all stages of dementia on their Qol. The studies were based on narrative and semi-structured interviews and focus group interviews, the latter partly supported by music and art therapy.

**Conclusions**

The meta-synthesis will contribute to the theoretical development of the concept of Qol of people with dementia. The resulting model of factors which affect dementia-specific Qol will provide a framework for the development, adaption and validation of dementia-specific Qol measurements and development of psychosocial nursing interventions.

### S3 Fidelity of dementia specific case conferences in nursing homes – Results of a process evaluation conducted parallel to a stepped- wedged cluster randomized controlled trial

#### Daniela Holle^1,2^, Rabea Graf^1^, Ute Rosier^1^, Sven Reuther^1,2^, Martina Roes^1,2^, Margareta Halek^1,2^

##### ^1^German Center of Neurodegenerative Diseases (DZNE), Witten, 58453 Witten, Germany; ^2^School of Nursing Science, Witten/Herdecke University, Witten, 58453 Witten, Germany

###### **Correspondence:** Daniela Holle (daniela.holle@dzne.de)

**Background**

Fidelity can act as a potential mediator of the relationship between interventions and their intended outcome. Within the FallDem-study the fidelity of dementia-specific case conferences (DSCC) was assessed as part of a process evaluation to explain the effects of DSCC on residents’ challenging behavior (primary outcome). Fidelity was assessed both in relation to the core elements of the DSCC (duration, frequency, process- and role structure, location) and potential adaptations, as well as the reach of the target populations (residents and nursing staff).

**Materials and methods**

Fidelity of DSCC was evaluated during the entire trial period using quantitative and qualitative methods. Standardized protocols of DSCC, attendance lists and written documentations of interventionists (trainers) as well as semi-structured interviews with participants of DSCC were conducted. Quantitative data were analysed using descriptive statistics, qualitative data were analysed using documentary analysis and content analysis. The concept of DSCC as well as the curriculum of delivering DSCC provided the basis for a systematic coding scheme for qualitative analysis.

**Results**

Results indicate a moderate fidelity of the core elements of DSCC. While the duration, role structure and location of DSCC was mostly followed, the frequency of DSCC was reduced by more than one third and adaptations were made regarding the process structure of DSCC. The reach of the target population indicates that 57% of expected residents (112/192) directly received the intervention, whereas the reach of the nursing staff was good, but characterized by a high turnover and limited continuity during the 7 months of intervention phase.

**Conclusion**

The moderate fidelity of DSCC as well as the high turnover and limited continuity of nursing staff might be influencing factors on the effects of DSCC on residents’ challenging behavior and should be carefully considered in the overall interpretation of the FallDem study results. A detailed analysis of the reasons for the moderate fidelity and the adaptations will be the next step of analysis.

**Trial registration**

Current Controlled Trials identifier: ISRCTN20203855, registered on 10^th^ July 2013.

**References**

1. Hasson H. Intervention Fidelity in Clinical Trials. In: Richards DA, Rahm Hallberg, I, editors. Complex Interventions in Health. Volume 1 1^st^ edition. London And New York: Routledge; 2015.232-238.

2. Holle D, Roes M, Buscher I, Reuther S, Müller R, Halek M. Process evaluation of the implementation of dementia-specific case conferences in nursing homes (FallDem): study protocol for a randomized controlled trial. Trials. 2014; 15:485. doi:10.1186/1745-6215-15-485.

3. Reuther S, Holle D, Buscher I, Dortmann O, Müller R, Bartholomeyczik S, Halek M. Effect evaluation of two types of dementia-specific case conferences in German nursing homes (FallDem) using a stepped-wedge design: study protocol for a randomized controlled trial. Trials. 2014*;* 15: 319, doi:10.1186/1745-6215-15-319.

### S4 A nurse-led rehabilitation programme (the ProBalance Programme) improves balance and reduces fall risk of community-dwelling older adults: a randomised controlled trial

#### Bruna R. Gouveia^1,2^, Helena G. Jardim^3^; Maria M. Martins^4^; Duarte L. Freitas^5^; José A. Maia^6^; Debra J. Rose^7^; Élvio R. Gouveia^5^

##### ^1^Saint Joseph of Cluny Higher School of Nursing, Funchal, 9050-535, Portugal; ^2^Institute of Biomedical Sciences Abel Salazar, University of Porto, Porto, 4050-313, Portugal; ^3^Higher School of Health, University of Madeira, Funchal, 9000-082, Portugal; ^4^Higher School of Nursing of Porto, Porto, 4200-072, Portugal; ^5^Dept of Physical Education and Sports, University of Madeira, Funchal, 9000-082, Portugal; ^6^Faculty of Sport, University of Porto, Porto, 4200-450, Portugal; ^7^Division of Kinesiology and Health Science, California State University Fullerton, Fullerton, California, 92831-3599, USA

###### **Correspondence:** Bruna R. Gouveia (bgouveia@esesjcluny.pt)

**Background**

Balance and fall risk are important outcomes in rehabilitation nursing care of older people.

This study aims to assess the effect of the ProBalance Programme on balance and fall risk of community-dwelling older people from Madeira, Portugal.

**Materials and methods**

A single-blind, randomised controlled trial (RCT) included community-dwelling older people (65-85 yrs) with balance impairments (n = 177). Participants were randomly allocated to an intervention group (IG; n = 27) or a wait-list control group (CG; n = 25). The intervention included gait, balance, functional training, strengthening, flexibility, and 3D training. The group-based intervention was administered by a rehabilitation nurse over 12 weeks (90-minute sessions, twice/week). The main outcome was balance, assessed by the Fullerton Advanced Balance (FAB) scale. Increases in FAB scores are associated to decreases in fall risk. The time points for assessment were at zero (pre-test), 12 (post-test), and 24 weeks (follow up).

**Results**

A trend for higher FAB scale scores in the IG was found, with significantly higher increases from the pre-test to the post-test, compared to the CG, which showed a significant decrease. At follow-up, a significant decrease was also found in the IG. There was a significant interaction of group with time (*F*(2,42) = 27.89, *p* < .001, η_p_^2^ = .57) and a main effect for time (*F*(2,43) = 3.76, p = .03, η_p_^2^ = .15), with both groups showing changes in the mean FAB scale scores across the three time periods. A significant main effect comparing the two groups (*F*(1,43) = 21.90, p < .001, η_p_^2^ = .34) confirmed a positive effect of the intervention.

**Conclusions**

This study demonstrated the ProBalance’s efficacy in improving balance and reducing fall risk of older people with balance impairment, immediately after the intervention. A decline in balance was observed for the IG after a period of no intervention.

**Trial Registration**

ACTRN12612000301864.

### S5 Addressing methodological issues in nursing outcomes research: RN4CAST’s contribution

#### Luk Bruyneel^1^, Emmanuel Lesaffre^2^, Walter Sermeus^1^

##### ^1^Leuven Institute for Healthcare Policy, KU Leuven – University of Leuven, 3000, Leuven, Belgium; ^2^Leuven Biostatistics and Statistical Bioinformatics Centre (L-BioStat), KU Leuven – University of Leuven, 3000, Leuven, Belgium

###### **Correspondence:** Luk Bruyneel (luk.bruyneel@med.kuleuven.be)

**Background**

Several methodological issues are rarely addressed in examining the association between the organization of nursing care and nurse wellbeing and patient outcomes. We identified a need for (1) a refined understanding of the similarity of effects across hierarchical levels (nursing units, hospitals), (2) an evaluation of the comparability of findings across groups and hierarchical levels, (3) a search for the optimum level of production in established associations, and (4) an examination of the mechanisms at play in these associations.

**Materials and methods**

The Registered Nurse Forecasting Study gathered data from 33731 nurses (wellbeing, organization of care) and thousands of patients (33731 satisfaction surveys, mortality data for 422730 patients). In a series of published and upcoming articles that exploit these unique data, multilevel structural equation modelling is applied to align theory and analysis and generate new evidence.

**Results**

In a first study we have shown that associations between work environment dimensions and nurse wellbeing are not homologous across multiple levels of analysis [1], which confirms the saying that a frequent shortcoming when ignoring the multilevel structure of the data is not what is misestimated, but what is not learned. Second, using complex Bayesian structural equation models on two occasions, for both the nurse survey data [2] and patient survey data [3] we have shown that survey questionnaire dimensions (e.g. nurse-physician relations for the nurse survey, discharge communication for the patient survey) must not be compared across groups or hierarchical levels without assessing measurement invariance, i.e. the extent to which respondents across groups perceive and interpret the content of survey instrument items in the same way. Third, in ongoing work drawing on recently introduced non-linear multilevel structural equation modelling techniques, we have shown that similar conclusions can be drawn from linear and non-linear models when it comes to the association between better quality of the nursing work environment and reduced feelings of burnout. Achieving excellence in nurse work environments pays off in terms of lower nurse-reported burnout rates. Fourth, in a multilevel moderated mediation analysis we have shown that nursing care left undone mediates the observed association between nurse staffing and patients’ experiences with care [4]. In ongoing work, we postulate that this is also the case for the association between nurse staffing and patient mortality.

**Conclusions**

The RN4CAST consortium has refined our understanding of the established associations between the organization of nursing care, nurse wellbeing, and patient outcomes.

**References**

1. Li B, Bruyneel L, Sermeus W, Van den Heede K, Matawie K, Aiken LH, et al. Group-level impact of work environment dimensions on burnout experiences among nurses: a multivariate multilevel probit model. Int J Nurs Stud. 2013; 2:281–91.

2. Bruyneel L, Li B, Squires A, Spotbeen S, Meuleman B, Lesaffre E, et al. Bayesian Multilevel MIMIC Modeling for Studying Measurement Invariance in Cross-group Comparisons. Med Care. 2014. [Epub aheah of print].

3. Orindi BO, Lesaffre E, Sermeus W, Bruyneel L. Impact of Cross-level Measurement Noninvariance on Hospital Rankings Based on Patient Experiences With Care in 7 European Countries. Med Care [Epub aheah of print].

4. Bruyneel L, Li B, Ausserhofer D, Lesaffre E, Dumitrescu I, Smith HL, et al. Organization of hospital nursing, provision of nursing care, and patient experiences with care in Europe. Med Care Res Rev. 2015; 72:643–64.

### S6 Causes & consequences of ‘Care Left Undone’

#### J. E. Ball^1,3^, L. Bruyneel^2^, L. Aiken^4^, C. Tishelman^3^, W. Sermeus^2^, P. Griffiths^1^

##### ^1^Faculty of Health Sciences, University of Southampton UK & National Institute for Health Research Collaboration for Leadership in Applied Health Research and Care (CLAHRC) Wessex, Southampton, UK; ^2^Leuven Institute for Healthcare Policy, KU Leuven – University of Leuven, 3000, Leuven, Belgium; ^3^Medical Management Centre (MMC), Department of Learning, Informatics, Management and Ethics (LIME), Karolinska Institutet (KI), Stockholm, Sweden; ^4^Center for Health Outcomes and Policy Research, University of Pennsylvania School of Nursing, Philadelphia, PA 19104 USA

###### **Correspondence:** J. E. Ball (jane.ball@soton.ac.uk)

**Background**

Research has found that low levels of RN staffing is associated with a higher risk of patients dying in hospital [1] and that necessary care is more likely to be left undone[2,3]. Care left undone, may be the mediator between RN staffing and patient outcomes.

**Aims**

Examine the factors associated with variation in care left undone and associations with patient outcomes.

**Methods**

Cross-sectional survey data (RN4Cast study - 26,516 RNs) on nurse staffing levels on acute hospital wards, nurse reports of care left undone, and routinely collected data on hospital related mortality are analysed. Mortality within 30 days of admission (adjusting for hospital and patient characteristics) for 422,730 patients following common surgeries in 300 hospitals in nine European countries examined. Multi-level regression models are used to assess the relationship between staffing, care left undone and mortality.

**Results**

Lower registered nurse staffing levels are associated with higher levels of care left undone and with an increased risk of patient death, even when other factors are controlled for.

**Conclusions**

Care left undone is an important consequence of low staffing levels, not just for the patient experience, but because it increases risk of patient.

Relevance for Nursing Science in Europe: The research has highlighted a cause of preventable patient deaths, rarely considered in patient safety literature.

**References**

1. Aiken LH, Sloane DM, Bruyneel L, Van den Heede K, Griffiths P, Busse R, et al. Nurse staffing and education and hospital mortality in nine European countries: a retrospective observational study. The Lancet. 2014;383(9931):1824–30.

2. Ball JE, Murrells T, Rafferty AM, Morrow E, Griffiths P. ‘Care left undone’ during nursing shifts: associations with workload and perceived quality of care. BMJ Quality & Safety. 2014;23(2):116–25.

3. Ausserhofer D, Zander B, Busse R, Schubert M, De Geest S, Rafferty AM, et al. Prevalence, patterns and predictors of nursing care left undone in European hospitals: results from the multicountry cross-sectional RN4CAST study. BMJ Qual Saf. 2014;23(2):126–35.

### S7 RN4CAST-CY and beyond: the RANCARE project

#### Evridiki Papastavrou, Panayiota Andreou

##### ^1^Department of Nursing, Cyprus University of Technology, Limassol, 3011, Cyprus

###### **Correspondence:** Evridiki Papastavrou (e.papastavrou@cut.ac.cy)

**Background**

The RN4CAST project is one of the most important contributions of Nursing in improving the health of European citizens by highlighting the links between education, performance and patient safety. Drawing from this experience, Cyprus will map and compare its findings within the European framework adding to the quality of patient care in the EU.

**Aim**

To explore the relationship between practice environment and specific patient and nurse outcomes in Cyprus.

**Methods**

Survey data, using the Practice Environment Scale of the Nursing Work Index (PES-NWI), job satisfaction and quality of care including work left undone, from 507 nurses in 14 general medical and surgical wards, across 5 Public Hospitals.

**Results**

The PES-NWI score was 2.36 (SD = 0.52), with males appearing more negative than females (2.25 vs. 2.42, p = 0.001). 44.5% of nurses reported no satisfaction with their work; 50.6% reported intention to leave in the next year. The most frequent activities left undone included comfort/talk with patients (58.3%), oral hygiene (56.9%) and educating patients and families (54.0%).

**Conclusions**

Nursing care left is prevalent across Europe including Cyprus. Building on this experience and the consistency of results internationally we aim to explore this issue further through a COST Action. The main objective of this Action is to facilitate discussion cross-nationally with implications for practice and professional development by advancing collaboration and networking and integrating different disciplines including nursing, moral philosophy and health care studies.

**Relevance for nursing science in Europe**

The COST Action CA15208 (RANCARE) http://www.cost.eu/COST_Actions/ca/CA15208 will facilitate a debate on the conceptualisation of care rationing and work left undone, the methodological challenges in investigating and monitoring the phenomenon, and enable research and policy synergies across Europe with an overall aim to address patient needs.

**Acknowledgements**

Special thanks to the participants and the collaborators assisting in the data collection and management.

### S8 The results of the RN4CAST study in Italy

#### Loredana Sasso^1^, Annamaria Bagnasco^1^, Milko P. Zanini^1^, Gianluca Catania^1^, Giuseppe Aleo^1^, Federico Spandonaro^2^, Giancarlo Icardi^1^, Roger Watson^3^, Walter Sermeus^4^

##### ^1^Department of Health Sciences, University of Genoa, Genoa 16132, Italy; ^2^Faculty of Economics, University of Rome Tor Vergata, Rome 00133, Italy; ^3^Faculty of Health and Social Care, University of Hull, Hull HU6 7RX, UK; ^4^Department of Public Health and Primary Care, KU Leuven, Leuven 3000, Belgium

###### **Correspondence:** Annamaria Bagnasco (annamaria.bagnasco@unige.it)

**Background**

The Nurse Forecasting Project in Europe (RN4CAST) [1] has already been conducted in 12 European countries, and it has shown that each additional surgical patient per nurse results in a 7% increase in the likelihood of dying within 30 days of admission, a 23% increase in burnout, and a 15% increase of job dissatisfaction [2]. Since Italy has 6.6 nurses per 1000 inhabitants in Italy, which is lower than the European average (11 nurses per 1000 inhabitants), we conducted the RN4CAST project also in Italy [3].

The main purpose of this study was to measure the impact of nurse staffing on patient outcomes, healthcare outcomes, and job satisfaction.

**Materials and methods**

RN4CAST survey including nurses, patients, and hospitals across Italy.

**Results**

The results will be officially presented at the RN4CAST@IT Dissemination Conference, which will be held on the 10^th^ June 2016 at the University of Genoa in Italy.

**Conclusions**

The RN4CAST project will bring about a significant change in Italy in terms of producing data that measure the impact of nurse staffing on patient outcomes, healthcare outcomes, and job satisfaction. In this way, health policy decision makers will have the information necessary to make informed decisions on nurse staffing levels, by considering nurses not just in terms of numbers, but also in relation to what type of patient outcomes nurse staffing levels are able to ensure in terms of reduced adverse events, complications, readmissions, and mortality, achieving the best cost-benefit ratio.

The RN4CAST project will enable to produce a database that can be used to measure the impact of nurse staffing and on patient outcomes, healthcare outcomes, and nurses’ job satisfaction. These results can be used to improve the accuracy of health workforce forecasting models and generate new approaches to ensure a more effective management of the nursing workforce across Europe.

**References**

1. Sermeus W, Aiken LH, Van den Heede K, Rafferty AM, Griffiths P, Moreno-Casbas MT, Busse R, Lindqvist R, Scott AP, Bruyneel L, Brostek T, Kinnunen J, Schubert M, Schoonhoven L, Zikos D, and RN4CAST consortium (2011). Nurse forecasting in Europe (RN4CAST): Rationale, design and methodology. *BMC Nurs*. 2011;10,6.

2. Aiken LH, Sloane DM, Bruyneel L, Van den Heede K, Griffiths P, Busse R, Diomidous M, Kinnunen J, Kòzka M, Lesaffre E, McHugh MD, Moreno-Casbas MT, Rafferty AM, Schwendimann R, Scott AP, Tishelman C, van Achterberg T & Sermeus W. 2014 Nurse staffing and education and hospital mortality in nine European countries: a retrospective observational study. *Lancet*. 2014; 383: 1824-1830.

3. Sasso L, Bagnasco A, Zanini M, Catania G, Aleo G, Santullo A, Spandonaro F, Icardi G, Watson R, Sermeus W. RN4CAST@ IT: why is it important for Italy to take part in the RN4CAST project? *J Adv Nurs.* 2015; 3(72): 485–487.

### S9 Reducing inappropriate use of indwelling urinary catheters in hospitals - a multiprofessional complex intervention? Results of a scoping review

#### Steffen Fleischer, Marion Burckhardt, Gabriele Meyer, Almuth Berg

##### Institute of Nursing and Health Sciences, Medical Faculty, Martin Luther University Halle-Wittenberg, Halle (Saale), 06112, Germany

###### **Correspondence:** Steffen Fleischer (steffen.fleischer@medizin.uni-halle.de)

**Background**

Indwelling urinary catheters go along with high risk of urinary tract infections. Clinical guidelines therefore recommend restrictive catheter use in hospitals. However, interventions to reduce indwelling urinary catheters in hospitals are challenging.

Several systematic reviews identified promising approaches mostly as part of intervention bundles. It remains unclear how intervention components should actually be implemented.

Therefore, we performed a comprehensive synthesis of existing evidence and aimed to identify and describe appropriate intervention components to reduce the use of indwelling urinary catheters in acute care hospitals, and to identify and describe implementation strategies associated with these intervention components.

**Methods**

We performed a scoping review with the following concepts of interest: interventions to reduce indwelling urinary catheters in hospitals, their theoretical foundation and drivers enabling implementation.

The systematic search was conducted in five consecutive steps from July 2015 to February 2016 and covered the Cochrane Library, Medline, Cinahl, and guideline databases. Primary research articles (published since 2008), systematic reviews, and clinical guidelines (updated at the latest in 2010) in German or English language were included (n = 70 publications).

To systemize implementation strategies for intervention components we used implementation drivers related to organization and staff competency according to the Active Implementation Framework.

**Results**

Identified core intervention components were defined indications for indwelling urinary catheters, reminder systems, and stop orders.

On the organizational level they are flanked by institutionalized guidelines addressing responsibilities and processes, integrating the program’s components in electronic medical records, and performance assessment and feedback. On the staff competency level the interventions are accompanied by various training modalities as well as deployment of staff with special responsibilities and authorization. Consideration of multiprofessional stakeholders’ view and circumstances by assessment of knowledge, barriers and needs was used to model an intervention and to support the implementation process.

The intervention components can be justified in a satisfactory manner by a range of guideline recommendations and systematically assessed evidence of mainly observational trials.

**Conclusions**

The identified intervention components and implementation strategies are suitable to model a complex intervention to reduce catheters in hospitals. Many of the components involve multiprofessional aspects. In particular, collaboration of physicians and nurses seems to be mandatory.

Limitations exist in the underlying evidence for some components due to used study designs in primary research.

### S10 The Patient Participation Culture Tool for healthcare workers (PaCT-HCW) on general hospital wards: a four-phased development and psychometric validation study

#### Ann Van Hecke^1,2^, Simon Malfait^1,2^, Johan Van Daele^2^, Kristof Eeckloo^1,2^

##### ^1^Faculty of Medicine and Health Sciences, University Centre for Nursing and Midwifery, Ghent University, 9000, Ghent, Belgium; ^2^Ghent University Hospital, 9000 Ghent, Belgium

###### **Correspondence:** Ann Van Hecke (Ann.VanHecke@UGent.be)

**Background**

Patient participation is essential to improve the health of European citizens in the 21st century. It reduces communication errors, increases patient empowerment, and is associated with positive health. The initiating behavior of healthcare workers to stimulate patient participation and their willingness to share their responsibilities and power with the patient is paramount. As behavior is embedded in a ward’s culture, insight in the patient participation culture on hospital wards is a necessity. Currently, little research on this topic is published and no validated tool is available to measure this culture on hospital wards. The aim of this study is to develop and validate the Patient Participation Culture Tool for healthcare workers (PACT-HCW), which measures the healthcare worker-related determinants of and effective communication in patient participation from the healthcare worker’s perspective.

**Materials and methods**

A four phased validation study was conducted. The construct of the PaCT-HCW was based on the ‘conceptual model of patient participation in error prevention’ [1] and the ‘comprehensive model of patient involvement’ [2], and focus interviews. During the instrument development phase, the identified components from the models were elaborated by use of literature. The content of the PaCT-HCW was validated by a Delphi procedure and 3 pilot studies. The PACT-HCW was psychometric evaluated by use of an exploratory factor analysis and by calculating the internal consistency.

**Results**

Fifteen general and university hospitals took part. Surgery, general medicine, long-term revalidation, geriatric and maternal care units were included. The respondents had to be healthcare workers, with hands-on patient contact, who worked on the same ward for more than six months. The tool was completed by 1329 respondents on 163 wards (Table 1).

**Table 1:** **(abstract S10).** Characteristics of the participants

Variable	n	%	Variable	n	%
Gender			Managerial position		
Male	241	18.13	Yes	237	17.83
Female	1088	81.87	No	1092	82.17
Profession			Age		
Nurse	873	65.69	<25 years	108	8.13
Midwife	122	9.18	25-34 years	402	30.25
Physician	144	10.84	35-44 years	331	24.91
Paramedical prof.	146	10.99	45-55 years	344	25.88
Nurse assistant	44	3.31	> 55 years	144	10.84
Education			Job time		
Secondary school	37	2.78	< 50%	115	8.65
Undergraduate	315	23.70	50-99%	449	33.78
Bachelor	723	54.40	100%	765	57.56
Master or higher	254	19.11			
Employment on the ward					
< 1 year	92	6.92			
≥ 1 year	1237	93.08			

The model showed high sampling adequacy (Kaiser-Meyer-Olkin Measure = 0.905) and the Bartlett’s test of sphericity was significant (χ = 15082.47; df = 1485; p < 0.001). The exploratory factor analysis identified eight components, explaining 49.88% of the variance (Table 2). The eight components of the PACT-HCW showed high internal consistency (Table 2).

**Table 2:** **(abstract S10).** An overview of the components with the explained variances and Cronbach’s α

Components	Nr of items	Nr of respondents	Scale mean	Standard deviation	Interitem correlations	% of variances	Cumulative %	Cronbach’s α	α if item deleted	Armor’s θ
Competence	3	1329	10.02	1.51	0.49-0.67	3.50	3.50	0.82	0.80	0.82
Support	8	1329	22.87	4.06	0.33-0.75	7.23	10.73	0.83	0.85	0.89
Perceived lack of time	3	1329	8.74	1.73	0.30-0.69	2.40	13.13	0.67	0.81	0.76
Information sharing and dialogue	18	831	48.76	10.74	0.31-0.75	13.34	26.47	0.93	0.93	0.93
Factual questions	5	1329	16.67	2.55	0.58-0.70	8.25	34.72	0.90	0.88	0.94
Challenging questions	4	1329	13.45	2.28	0.55-0.66	5.69	40.41	0.86	0.83	0.90
Notifying questions	4	1329	14.15	1.86	0.53-0.65	4.72	45.13	0.85	0.83	0.90
Acceptance of a new role	7	1329	18.98	3.02	0.29-0.57	4.75	49.88	0.70	0.71	0.77
Total	52	831	152.91	16.70	0.22-0.75	49.88	49.88	0.92	0.92	0.92

**Conclusion**

The PaCT-HCW is a thoroughly developed tool with high psychometric values. Hence, the PACT-HCW offers a valuable and nuanced view on the healthcare worker’s side of patient participation. Moreover, by creating more insight in the patient participation culture on hospital wards, the PaCT-HCW is useful for researchers, nursing managers and clinicians to undertake actions. The information from the PaCT-HCW allows the development of tailored interventions to enhance patient participation on the hospital wards.

**References**

1. Longtin Y, Sax H, Leape L, Sheridan S, Donaldson L, Pittet D. Patient participation: Current knowledge and applicability to patient safety. Mayo Clinic. Proc. 2010;53–62.

2. Tambuyzer E, Pieters G, Van Audenhove C. Patient involvement in mental health care: One size does not fit all. Health Expect. 2011;17(1):138–150.

### S11 Development and feasibility of ‘G-COACH: geriatric co-management for cardiology patients in the hospital’ – A case example of a mixed methods research project

#### Mieke Deschodt^1,2,3^, Bastiaan Van Grootven^1^, Koen Milisen^1,4^, Johan Flamaing^2,4^

##### ^1^Department of Public Health and Primary Care, University of Leuven KU Leuven, Leuven, Belgium; ^2^Department of Clinical and Experimental Medicine, University of Leuven KU Leuven, Leuven, Belgium; ^3^Department of Public Health, University of Basel, Basel, Switzerland; ^4^Division of Geriatrics, University Hospitals Leuven, Leuven, Belgium

###### **Correspondence:** Mieke Deschodt (mieke.deschodt@kuleuven.be)

**Background**

Older patients are at increased risk of adverse outcomes because of inappropriate hospital care. There is an urgent need to explore innovative care models that are able to sustain or improve healthcare outcomes for older adults as the current number of geriatricians and geriatric wards are inadequate to cope with the ageing population. G-COACH aims to test the efficacy of a geriatric co-management care model in cardiac patients. Geriatric co-management is characterized by collaboration and shared decision-making between the interdisciplinary geriatric team and treating physician.

**Materials and methods**

G-COACH is a multi-phase mixed methods research project incorporating: 1) a systematic literature review to identify structure, process and outcome variables; 2) a two-round international Delphi study to determine appropriate and feasible indicators; 3) a pre-post intervention study to test the efficacy of a geriatric co-management intervention based on the selected indicators; and 4) a qualitative evaluation using focus groups and individual interviews with core members of the co-management team in order to evaluate the feasibility of a large scale implementation study.

**Results**

Data extraction is currently being conducted on 44 publications included in the review. Results of the review and meta-analysis are expected in April. Fifteen US and 16 European experts participating in the first Delphi round, evaluated 5 structure, 12 process and 12 outcome indicators. Results of the final round are expected by the end of March.

**Conclusion**

A complex geriatric co-management intervention will be developed using program theory in collaboration with local stakeholder groups (May 2016), and subsequently evaluated in a before-after study (September 2016 – 2017). G-COACH is an exemplary case project that demonstrates the added value of using the MRC framework and incorporating a mixed methods approach.

**Acknowledgements**

This research is supported by the KU Leuven Research Council (REF 22/15/028; G-COACH). The investigators retained full independence in the conduct of this research. This abstract is published on behalf of the G-COACH consortium.

### S12 DEcision Coaching In Multiple Sclerosis (DECIMS) – pilot randomised controlled trial and process evaluation

#### Anne C Rahn^1,2^, Sascha Köpke^3^, Imke Backhus^2^, Jürgen Kasper^4^, Anna Krützelmann^2^, Ingo Kleiter^5^, Ingrid Mühlhauser^1^, Christoph Heesen^2^

##### ^1^Unit of Health Sciences and Education, University of Hamburg, Hamburg, 20146, Germany; ^2^Institute for Neuroimmunology and Multiple Sclerosis, Department of Neurology, University Medical Centre Hamburg-Eppendorf, Hamburg, 20246, Germany; ^3^Nursing Research Unit, Institute of Social Medicine and Epidemiology, University of Lübeck, Lübeck, Schlesweig-Holstein, 23538, Germany; ^4^Department of Health and Care Sciences, University of Tromsø, Tromsø, 9019, Norway; ^5^Ruhr-University Bochum, St. Josef Hospital, Bochum, 44791, Germany

###### **Correspondence:** Anne C. Rahn (a.rahn@uke.de)

**Background**

The “nurse decision coach model” was developed to redistribute health professionals’ tasks in supporting immunotherapy decision making processes by people with multiple sclerosis (PwMS), following the principles of shared decision making.

**Aims**

The pilot study aimed to test the randomisation procedure and to gather data on feasibility to inform the main trial.

**Methods**

PwMS (age ≥ 18 y) with suspected or relapsing-remitting MS facing an immunotherapy treatment decision were recruited in two German MS centres between March 2014 and June 2015. PwMS were randomised to the intervention (IG) or control group (CG). PwMS in IG received decision coaching by a trained nurse. PwMS in the CG received care as usual. Both groups had access to an evidence-based online information platform. Nurses were not blinded to group assignment. PwMS and physicians were kept blinded. The primary outcome was ‘informed choice’ after six months, a multi-dimensional measure including the sub-dimensions risk knowledge (questionnaire assessed after 14 days), attitude concerning immunotherapy (one question assessed after physician consultation), and therapy uptake (survey after six months).

**Results**

In total 73 PwMS were included, 38 in the IG and 35 in the CG. Groups were comparable at baseline. Data of 49 PwMS were available for the primary endpoint calculation. No significant difference was shown with 15 of 29 (52%) PwMS in the IG achieving informed choice after six months compared with 6 of 20 (30%) in the CG. Analysis of coaching videos showed good involvement of PwMS in shared decision making. Process evaluation data showed a positive response towards the programme.

**Conclusions**

The innovative approach of delegating treatment information provision to trained nurses has the potential to change current doctor-focussed practice in Germany.

**Trial registration**

Current Controlled Trials ISRCTN37929939.

